# Supplementary material for: Clinical evaluation of a non-purified direct molecular assay for the detection of Clostridioides difficile toxin genes in stool specimens
Source: PLoS One. 2020 Jun 3;15(6):e0234119. doi: 10.1371/journal.pone.0234119 (PMC7269250; doi:10.1371/journal.pone.0234119)
Supplement: S1 File — (DOCX) [file pone.0234119.s001.docx]

**Clinical Evaluation of a Non-purified Direct Molecular Assay for the Detection of *Clostridioides difficile* Toxin Genes in Stool Specimens**

Toshinori Hara^1,2^#, Hiromichi Suzuki^3,^ , Tadatomo Oyanagi^4^, Norito Koyanagi^5^, Akihito Ushiki^6^, Naoki Kawabata^7^, Miki Goto^8^, Yukio Hida^9^, Yuji Yaguchi^10^, Kiyoko Tamai^10^, Shigeyuki Notake^11^, Yosuke Kawashima^12^, Akio Sugiyama^12^, Keiichi Uemura^5^, Seiya Kashiyama^1,2^, Toru Nanmoku^8^, Satoshi Suzuki^13^, Hiroshi Yamazaki^7^, Hideki Kimura^9^, Hiroyuki Kunishima^14^, Hiroki Ohge^15^

1. Section of Clinical Laboratory, Department of Clinical Practice and Support, Hiroshima University Hospital, Hiroshima, Japan

2. Division of Clinical Laboratory Medicine, Hiroshima University Hospital, Hiroshima, Japan

3. Division of Infectious Diseases, Department of Medicine, Tsukuba Medical Center Hospital, Ibaraki, Japan

4. Department of Clinical Laboratory, St. Marianna University School of Medicine Hospital, Kanagawa, Japan

5. Department of Clinical Laboratory, Chutoen General Medical Center, Shizuoka, Japan

6. Department of Clinical Laboratory, Tone Chuo Hospital, Gunma, Japan

7. Department of Clinical Laboratory, Tsuruga Municipal Hospital, Fukui, Japan

8. Department of Clinical Laboratory, University of Tsukuba Hospital, Ibaraki, Japan

9. Department of Clinical Laboratory, University of Fukui Hospital, Fukui, Japan

10. Miroku medical laboratory Inc., Nagano, Japan

11. Department of Clinical Laboratory, Tsukuba Medical Center Hospital, Ibaraki, Japan

12. Diagnostic System Department, TOYOBO Co., Ltd., Osaka, Japan

13. Division of General Medicine, Tone Chuo Hospital, Gunma, Japan

14. Department of Infectious Diseases, St. Marianna University School of Medicine, Kanagawa, Japan

15. Department of Infectious diseases, Hiroshima University Hospital, Hiroshima, Japan

#Address correspondence to Toshinori Hara, t2128@hiroshima-u.ac.jp

Correspondence

Toshinori Hara

Section of Clinical Laboratory, Department of Clinical Practice and Support, Hiroshima University Hospital, Hiroshima, Japan

Division of Clinical Laboratory Medicine, Hiroshima University Hospital, Hiroshima, Japan

Hiroshima University Hospital

1-2-3 Kasumi, Minami-ku, hiroshima 734-8551, Japan

Tel.: +81-(82)-257-5546

Fax: +81-(82)-257-5546

E-mail: t2128@hiroshima-u.ac.jp

Supplement file 1：A list of the evaluations of each stool sample using C. DIFF QUIK CHEK COMPLETE, GENECUBE C. difficile assay, BD MAX Cdiff assay, and toxigenic culture

|  | *C.DIFF* QUIK CHEK | | GENECUBE | BD MAX | Toxigenic culture | |
| --- | --- | --- | --- | --- | --- | --- |
|  | GDH | Toxin | *tcdB* | *tcdB* | *C.difficile* | toxin |
| 1 | - | - | - | - | - | - |
| 2 | + | + | + | + | + | + |
| 3 | - | - | - | - | - | - |
| 4 | - | - | - | - | - | - |
| 5 | + | - | - | - | + | - |
| 6 | - | - | - | - | - | - |
| 7 | - | - | - | - | - | - |
| 8 | + | - | + | + | + | + |
| 9 | + | + | + | + | + | + |
| 10 | - | - | - | - | - | - |
| 11 | - | - | - | - | - | - |
| 12 | - | - | - | - | - | - |
| 13 | + | - | + | + | + | + |
| 14 | - | - | - | - | - | - |
| 15 | - | - | - | - | + | - |
| 16 | - | - | - | - | + | - |
| 17 | - | - | - | - | - | - |
| 18 | + | - | - | - | + | - |
| 19 | + | + | + | + | + | + |
| 20 | + | + | + | + | + | + |
| 21 | - | - | - | - | - | - |
| 22 | - | - | - | - | - | - |
| 23 | - | - | - | - | - | - |
| 24 | - | - | - | - | - | - |
| 25 | - | - | - | - | - | - |
| 26 | - | - | - | - | - | - |
| 27 | - | - | - | - | - | - |
| 28 | - | - | - | - | - | - |
| 29 | - | - | - | - | - | - |
| 30 | - | - | - | - | - | - |
| 31 | - | - | - | - | - | - |
| 32 | - | - | - | - | + | + |
| 33 | - | - | - | - | - | - |
| 34 | - | - | - | - | - | - |
| 35 | - | - | - | - | - | - |
| 36 | - | - | - | - | - | - |
| 37 | - | - | - | - | - | - |
| 38 | - | - | - | - | - | - |
| 39 | + | - | - | - | + | - |
| 40 | - | - | - | - | - | - |
| 41 | - | - | - | - | - | - |
| 42 | - | - | - | - | - | - |
| 43 | - | - | - | - | - | - |
| 44 | - | - | - | - | + | - |
| 45 | + | - | + | + | + | + |
| 46 | - | - | - | - | - | - |
| 47 | - | - | - | - | - | - |
| 48 | - | - | - | - | - | - |
| 49 | - | - | - | - | - | - |
| 50 | - | - | - | - | - | - |
| 51 | - | - | - | - | - | - |
| 52 | - | - | - | - | - | - |
| 53 | - | - | - | - | - | - |
| 54 | - | - | - | - | - | - |
| 55 | - | - | - | - | - | - |
| 56 | - | - | - | - | - | - |
| 57 | - | - | - | - | - | - |
| 58 | - | - | - | - | - | - |
| 59 | - | - | - | - | - | - |
| 60 | - | - | - | - | - | - |
| 61 | + | + | + | + | + | + |
| 62 | - | - | - | - | - | - |
| 63 | + | - | - | - | + | - |
| 64 | + | - | + | - | + | + |
| 65 | + | - | + | + | + | + |
| 66 | - | - | - | - | - | - |
| 67 | - | - | - | - | - | - |
| 68 | - | - | - | - | - | - |
| 69 | - | - | - | - | - | - |
| 70 | + | - | + | + | + | + |
| 71 | - | - | - | - | - | - |
| 72 | - | - | - | - | - | - |
| 73 | + | - | + | + | + | + |
| 74 | + | - | + | + | + | + |
| 75 | + | - | - | - | + | - |
| 76 | - | - | - | - | - | - |
| 77 | + | - | + | - | + | + |
| 78 | - | - | - | - | - | - |
| 79 | - | - | - | - | - | - |
| 80 | - | - | - | - | - | - |
| 81 | - | - | - | - | - | - |
| 82 | - | - | - | - | - | - |
| 83 | - | - | - | - | - | - |
| 84 | - | - | - | - | - | - |
| 85 | - | - | - | - | - | - |
| 86 | - | - | - | - | - | - |
| 87 | - | - | - | - | - | - |
| 88 | + | - | + | + | + | + |
| 89 | + | - | + | + | + | + |
| 90 | - | - | - | - | - | - |
| 91 | + | - | - | - | + | - |
| 92 | - | - | - | - | - | - |
| 93 | - | - | - | - | - | - |
| 94 | - | - | - | - | - | - |
| 95 | - | - | - | - | - | - |
| 96 | + | - | - | - | + | - |
| 97 | + | + | + | + | + | + |
| 98 | + | - | - | - | + | - |
| 99 | - | - | - | - | - | - |
| 100 | + | - | + | + | + | + |
| 101 | - | - | - | - | + | - |
| 102 | - | - | - | - | - | - |
| 103 | - | - | - | - | - | - |
| 104 | + | - | + | + | + | + |
| 105 | - | - | - | - | - | - |
| 106 | - | - | - | - | - | - |
| 107 | - | - | - | - | - | - |
| 108 | - | - | - | - | - | - |
| 109 | + | - | - | - | + | - |
| 110 | - | - | - | - | - | - |
| 111 | - | - | - | - | - | - |
| 112 | - | - | - | - | - | - |
| 113 | + | + | + | + | + | + |
| 114 | + | + | - | - | - | - |
| 115 | - | - | - | - | - | - |
| 116 | - | - | - | - | - | - |
| 117 | - | - | - | - | - | - |
| 118 | + | + | + | + | + | + |
| 119 | - | - | - | - | - | - |
| 120 | - | - | - | - | - | - |
| 121 | - | - | - | - | - | - |
| 122 | - | - | - | - | - | - |
| 123 | + | - | + | + | + | + |
| 124 | - | - | - | - | - | - |
| 125 | - | - | - | - | + | + |
| 126 | + | - | + | + | + | + |
| 127 | - | - | - | - | - | - |
| 128 | - | - | - | - | - | - |
| 129 | - | - | - | - | - | - |
| 130 | - | - | - | - | - | - |
| 131 | - | - | - | - | - | - |
| 132 | - | - | - | - | - | - |
| 133 | - | - | - | - | - | - |
| 134 | - | - | - | - | - | - |
| 135 | - | - | - | - | - | - |
| 136 | - | - | - | - | - | - |
| 137 | - | - | + | + | + | + |
| 138 | - | - | - | - | - | - |
| 139 | - | - | - | - | - | - |
| 140 | - | - | - | - | - | - |
| 141 | - | - | - | - | - | - |
| 142 | + | - | - | - | + | - |
| 143 | - | - | + | + | + | + |
| 144 | - | - | - | - | - | - |
| 145 | - | - | - | - | - | - |
| 146 | - | - | - | - | - | - |
| 147 | - | - | - | - | - | - |
| 148 | - | - | - | - | - | - |
| 149 | - | - | - | - | - | - |
| 150 | - | - | - | - | - | - |
| 151 | - | - | - | - | - | - |
| 152 | - | - | - | - | - | - |
| 153 | - | - | - | - | - | - |
| 154 | - | - | - | - | - | - |
| 155 | - | - | - | - | - | - |
| 156 | - | - | - | - | - | - |
| 157 | - | - | - | - | - | - |
| 158 | - | - | - | - | - | - |
| 159 | - | - | - | - | - | - |
| 160 | + | + | + | + | + | + |
| 161 | - | - | - | - | - | - |
| 162 | - | - | - | - | - | - |
| 163 | - | - | - | - | + | - |
| 164 | - | - | - | - | - | - |
| 165 | - | - | - | - | - | - |
| 166 | - | - | - | - | - | - |
| 167 | - | - | - | - | - | - |
| 168 | - | - | - | - | - | - |
| 169 | - | - | - | - | - | - |
| 170 | - | - | - | - | - | - |
| 171 | - | - | - | - | - | - |
| 172 | - | - | - | - | - | - |
| 173 | - | - | - | - | - | - |
| 174 | - | - | - | - | - | - |
| 175 | - | - | - | - | - | - |
| 176 | - | - | - | - | - | - |
| 177 | - | - | - | - | - | - |
| 178 | - | - | - | - | - | - |
| 179 | - | - | - | - | - | - |
| 180 | - | - | - | - | - | - |
| 181 | - | - | - | - | + | + |
| 182 | - | - | - | - | - | - |
| 183 | + | - | + | + | + | + |
| 184 | - | - | - | - | - | - |
| 185 | - | - | - | - | - | - |
| 186 | - | - | - | - | - | - |
| 187 | - | - | - | - | + | + |
| 188 | - | - | - | - | - | - |
| 189 | - | - | - | - | - | - |
| 190 | - | - | - | - | - | - |
| 191 | - | - | - | - | - | - |
| 192 | - | - | - | - | - | - |
| 193 | - | - | - | - | - | - |
| 194 | - | - | - | - | - | - |
| 195 | - | - | - | - | - | - |
| 196 | - | - | - | - | - | - |
| 197 | - | - | - | - | - | - |
| 198 | + | - | - | - | + | - |
| 199 | - | - | - | - | - | - |
| 200 | - | - | - | - | - | - |
| 201 | - | - | + | + | + | + |
| 202 | - | - | - | - | - | - |
| 203 | - | - | - | - | - | - |
| 204 | - | - | - | - | - | - |
| 205 | - | - | + | + | + | + |
| 206 | - | - | - | - | - | - |
| 207 | - | - | - | - | - | - |
| 208 | - | - | - | - | - | - |
| 209 | - | - | - | - | - | - |
| 210 | - | - | - | - | - | - |
| 211 | + | + | + | + | + | + |
| 212 | + | + | + | + | + | + |
| 213 | - | - | - | - | - | - |
| 214 | - | - | + | + | + | + |
| 215 | - | - | - | - | - | - |
| 216 | - | - | - | - | - | - |
| 217 | - | - | - | - | - | - |
| 218 | - | - | - | - | - | - |
| 219 | - | - | - | - | - | - |
| 220 | - | - | - | - | - | - |
| 221 | - | - | - | - | - | - |
| 222 | - | - | - | - | - | - |
| 223 | - | - | - | - | - | - |
| 224 | - | - | - | - | - | - |
| 225 | - | - | - | - | - | - |
| 226 | - | - | - | - | - | - |
| 227 | - | - | - | - | - | - |
| 228 | - | - | - | - | - | - |
| 229 | + | - | + | + | + | + |
| 230 | - | - | - | - | - | - |
| 231 | + | + | + | + | + | + |
| 232 | - | - | - | - | - | - |
| 233 | + | + | + | + | + | + |
| 234 | - | - | - | - | - | - |
| 235 | - | - | - | - | - | - |
| 236 | + | - | - | - | + | - |
| 237 | + | - | + | + | - | - |
| 238 | - | - | - | - | - | - |
| 239 | - | - | - | - | - | - |
| 240 | - | - | - | - | - | - |
| 241 | - | - | - | - | - | - |
| 242 | + | - | + | + | + | + |
| 243 | - | - | - | - | - | - |
| 244 | - | - | - | - | - | - |
| 245 | - | - | - | - | - | - |
| 246 | - | - | - | - | - | - |
| 247 | - | - | - | - | - | - |
| 248 | - | - | - | - | - | - |
| 249 | + | - | - | - | + | - |
| 250 | - | - | - | - | - | - |
| 251 | - | - | - | - | - | - |
| 252 | - | - | - | - | - | - |
| 253 | - | - | - | - | - | - |
| 254 | - | - | - | - | + | - |
| 255 | - | - | - | - | - | - |
| 256 | - | - | + | + | + | + |
| 257 | - | - | - | - | - | - |
| 258 | - | - | - | - | - | - |
| 259 | - | - | - | - | + | - |
| 260 | + | - | - | - | + | - |
| 261 | - | - | - | - | - | - |
| 262 | - | - | - | - | - | - |
| 263 | - | - | - | - | - | - |
| 264 | - | - | - | - | - | - |
| 265 | - | - | + | + | + | + |
| 266 | - | - | - | - | - | - |
| 267 | - | - | - | - | - | - |
| 268 | - | - | - | - | - | - |
| 269 | - | - | - | - | - | - |
| 270 | - | - | - | - | - | - |
| 271 | - | - | - | - | - | - |
| 272 | - | - | - | - | - | - |
| 273 | - | - | - | - | - | - |
| 274 | - | - | - | - | - | - |
| 275 | - | - | - | - | - | - |
| 276 | + | - | - | - | + | - |
| 277 | - | - | - | - | - | - |
| 278 | - | - | - | - | - | - |
| 279 | - | - | - | - | - | - |
| 280 | - | - | - | - | - | - |
| 281 | - | - | - | - | - | - |
| 282 | - | - | - | - | - | - |
| 283 | + | - | + | + | + | + |
| 284 | - | - | - | - | - | - |
| 285 | - | - | - | - | - | - |
| 286 | + | - | - | - | + | - |
| 287 | - | - | - | - | - | - |
| 288 | + | - | - | - | - | - |
| 289 | - | - | - | - | - | - |
| 290 | - | - | - | - | - | - |
| 291 | - | - | - | - | - | - |
| 292 | - | - | - | - | - | - |
| 293 | - | - | - | - | - | - |
| 294 | - | - | - | - | - | - |
| 295 | - | - | - | - | - | - |
| 296 | - | - | - | - | - | - |
| 297 | - | - | - | - | - | - |
| 298 | - | - | - | - | + | + |
| 299 | - | - | - | - | - | - |
| 300 | + | + | + | + | + | + |
| 301 | - | - | - | - | - | - |
| 302 | - | - | - | - | - | - |
| 303 | - | - | - | - | - | - |
| 304 | - | - | - | - | - | - |
| 305 | - | - | - | - | - | - |
| 306 | + | - | + | + | + | + |
| 307 | - | - | - | - | - | - |
| 308 | - | - | - | - | - | - |
| 309 | - | - | - | - | - | - |
| 310 | - | - | - | - | - | - |
| 311 | - | - | - | - | - | - |
| 312 | - | - | - | - | - | - |
| 313 | + | - | + | - | + | + |
| 314 | + | + | + | + | + | + |
| 315 | - | - | - | - | - | - |
| 316 | - | - | - | - | - | - |
| 317 | - | - | - | - | + | + |
| 318 | - | - | - | - | - | - |
| 319 | - | - | - | - | - | - |
| 320 | - | - | - | - | - | - |
| 321 | - | - | - | - | - | - |
| 322 | - | - | - | - | - | - |
| 323 | - | - | - | - | - | - |
| 324 | - | - | - | - | - | - |
| 325 | - | - | - | - | - | - |
| 326 | - | - | - | - | - | - |
| 327 | + | - | + | + | + | + |
| 328 | - | - | - | - | - | - |
| 329 | - | - | - | - | - | - |
| 330 | - | - | + | + | - | - |
| 331 | - | - | - | - | - | - |
| 332 | - | - | - | - | - | - |
| 333 | - | - | - | - | - | - |
| 334 | - | - | - | - | - | - |
| 335 | - | - | - | - | - | - |
| 336 | - | - | + | + | + | + |
| 337 | - | - | - | - | + | - |
| 338 | - | - | - | - | - | - |
| 339 | - | - | - | - | - | - |
| 340 | - | - | - | - | - | - |
| 341 | - | - | - | - | - | - |
| 342 | - | - | - | - | - | - |
| 343 | - | - | - | - | - | - |
| 344 | - | - | - | - | - | - |
| 345 | - | - | - | - | - | - |
| 346 | - | - | - | - | - | - |
| 347 | - | - | - | - | - | - |
| 348 | - | - | - | - | - | - |
| 349 | - | - | - | - | - | - |
| 350 | - | - | - | - | - | - |
| 351 | - | - | - | - | - | - |
| 352 | - | - | - | - | + | + |
| 353 | + | - | + | + | - | - |
| 354 | - | - | - | + | - | - |
| 355 | - | - | - | - | - | - |
| 356 | - | - | - | - | - | - |
| 357 | + | - | - | - | - | - |
| 358 | - | - | - | - | + | + |
| 359 | - | - | - | - | - | - |
| 360 | - | - | - | - | - | - |
| 361 | - | - | - | - | - | - |
| 362 | + | - | + | + | + | + |
| 363 | - | - | - | - | - | - |
| 364 | - | - | - | - | - | - |
| 365 | - | - | - | - | - | - |
| 366 | - | - | - | - | - | - |
| 367 | + | - | + | + | + | + |
| 368 | - | - | - | - | - | - |
| 369 | - | - | - | - | - | - |
| 370 | - | - | - | - | - | - |
| 371 | - | - | - | - | - | - |
| 372 | - | - | + | + | - | - |
| 373 | - | - | - | - | - | - |
| 374 | + | - | + | + | + | + |
| 375 | - | - | - | - | - | - |
| 376 | - | - | - | - | - | - |
| 377 | + | - | + | + | + | + |
| 378 | - | - | - | - | + | - |
| 379 | - | - | - | - | - | - |
| 380 | - | - | - | - | + | + |
| 381 | + | - | + | + | + | + |
| 382 | - | - | - | - | - | - |
| 383 | + | + | + | + | + | + |
