# Supplementary material for: Clinical evaluation of a non-purified direct molecular assay for the detection of Clostridioides difficile toxin genes in stool specimens
Source: PLoS One. 2020 Jun 3;15(6):e0234119. doi: 10.1371/journal.pone.0234119 (PMC7269250; doi:10.1371/journal.pone.0234119)
Supplement: Attachment — Submitted filename: response to reviewers.docx [file pone.0234119.s002.docx]

Dear Editor and Reviewer

Thank you very much for reviewing our manuscript and offering valuable advice. We have addressed your comments with point-by-point responses, and revised the manuscript accordingly.

Reviewer #1: This is a competently performed evaluation of a new molecular assay for the detection of C. difficile *tcdB* gene. There are some specific issues that the authors should address.

1. The use of the terms "positive and negative concordance" rather than "sensitivity and specificity" which are terms likely to be both familar and better understood by the readers seems a odd construct. I understand that when they compared the two molecular assay the "concordance" construct might seem more appropriate to the authors. However when comparing the molecular test to toxigenic culture, a widely accepted *C. difficile* reference methods (although one that has its own short-comings), sensitivity and specificity does seem more appropriate.

Response: Thank you very much for your suggestion. We have changed the sentences as suggested.

2. ln 48-49: Since GENECUBE assay was only compared to the BD MAX assay, this is an overstatement. Please modify.

Response: Thank you very much for your suggestion. We have changed the text to show it was only compared with the BD MAX assay (line 47).

3. ln 102-4: Since QUIK CHECK complete is widely used diagnostically, it would be interesting to have compared the GENECUBE performance to it in a manner similar to what was done with BD MAX with toxigenic culture used as a reference method to resolve discrepant results.

Response: Thank you for this comment. C. DIFF QUIK CHEK COMPLETE was useful, especially for the analysis of discrepant results between molecular examination and toxigenic culture.

4. Ln 114-7: This seems a significant delay between sample collection and test performance. Perhaps a comment in the discussion is warranted?

Response: We agree that a delay would negatively affect the test performance of the BD MAX assay. We have added this point regarding the negative effect to the limitation section of discussion (lines 296-297).

5. ln 170-2: How was a "postive" test defined in the LOD portion of the study for the two molecular assays.

Response: The GENECUBE system and the BD MAX system automatically show the results of molecular analyses on a display when the assay evaluations are complete. We used the automatic analysis to determine “positive” and “negative” for the current evaluation (lines 133-135 and Table 2-c).

6. ln 262-6: One of the short-comings of NAAT assays for *C. difficile* is false positive when compared to toxigenic culture. These "false" positives are even more pronouced when cytotoxicity assays are used as reference methods. This point likely deserves a bit more discussion in your manuscript.

Response: Thank you very much for your review and suggestions. In this study, GDH was also positive in two of the four culture-negative stool samples. We think that toxigenic culture with the alcohol shock method is a highly sensitive method for the detection of toxigenic *C. difficile*; however, some culture-negative stool samples can be detected by molecular examination. We have added new references and sentences to the discussion section as red text (lines 266-272).

7. Ln 281: To be clear, the two assays to which you are referring are the LIAT and Xpert assays. Correct?

Response: Thank you for this question. You are correct, the two assays are the cobas Liat Cdiff assay and Xpert *C. difficile* assay. We revised the text from “the two assays” to the “Liat Cdiff and Xpert *C. difficile* assays”.

To the editorial Office

1. Please ensure that your manuscript meets PLOS ONE's style requirements, including those for file naming. The PLOS ONE style templates can be found at

https://journals.plos.org/plosone/s/file?id=wjVg/PLOSOne_formatting_sample_main_body.pdf and

https://journals.plos.org/plosone/s/file?id=ba62/PLOSOne_formatting_sample_title_authors_affiliations.pdf

Response: Thank you very much for this suggestion. We have modified the style requirements by referring to the above PDF.

2. PLOS requires an ORCID iD for the corresponding author in Editorial Manager on papers submitted after December 6th, 2016. Please ensure that you have an ORCID iD and that it is validated in Editorial Manager. To do this, go to ‘Update my Information’ (in the upper left-hand corner of the main menu), and click on the Fetch/Validate link next to the ORCID field. This will take you to the ORCID site and allow you to create a new iD or authenticate a pre-existing iD in Editorial Manager. Please see the following video for instructions on linking an ORCID iD to your Editorial Manager account: https://www.youtube.com/watch?v=_xcclfuvtxQ

Response: The corresponding author has obtained an ORCID iD.

3. The [PLOS Data policy](https://jpn01.safelinks.protection.outlook.com/?url=http%3A%2F%2Fwww.plosone.org%2Fstatic%2Fpolicies.action%23sharing&data=02%7C01%7Ct2128%40hiroshima-u.ac.jp%7C42a7fb18b7d04d3b68d908d7f1bf55b1%7Cc40454ddb2634926868d8e12640d3750%7C1%7C1%7C637243676393001700&sdata=K2HocDYeAqI8RSGElqhc%2BY6qVAJMkyYVlAR2K38t%2F8Y%3D&reserved=0) requires authors to make all data underlying the findings described in their manuscript fully available without restriction, with rare exception (please refer to the Data Availability Statement in the manuscript PDF file). The data should be provided as part of the manuscript or its supporting information, or deposited to a public repository. For example, in addition to summary statistics, the data points behind means, medians and variance measures should be available. If there are restrictions on publicly sharing data—e.g. participant privacy or use of data from a third party—those must be specified.

Response: We added a supporting information file to show all of the individual data
